# Years of life lost in patients with a false-negative diagnosis of primary melanoma. A prospective study of the German Central Malignant Melanoma Registry involving 9063 patients over 28 years

**DOI:** 10.1038/s41467-026-74443-9

**Published:** 2026-07-02

**Authors:** Claus Garbe, Ulrike Keim, Teresa Amaral, Paolo A. Ascierto, Jürgen Bauer, Reinhard Dummer, Alexander M. M. Eggermont, Thomas K. Eigentler, Lukas Flatz, Stephan Forchhammer, Andrea Forschner, Sara Gandini, Jeffrey E. Gershenwald, Axel Hauschild, Christoph Hoeller, John M. Kirkwood, Celeste Lebbe, Georgina V. Long, Paul Lorigan, Jason J. Luke, Friedegund Meier, Gisela Metzler, Paul Nathan, Michael A. Postow, Caroline Robert, Martin Röcken, Dirk Schadendorf, Benjamin Weide, David Whiteman, Ulrike Leiter, Peter Martus

**Affiliations:** 1 Center for Dermatooncology, Department of Dermatology, University Hospital of Tuebingen, Tuebingen, Germany; 2 Practice Dermatology in Stuttgart, Stuttgart, Germany; 3 Institute for Health Sciences, University of Tuebingen, Tuebingen, Germany; 4 Department of Skin Cancer, Cancer Immunotherapy and Development Therapeutics. Instituto Nazionale Tumouri IRCCS Fondazione G. Pascale, Napoli, Italy; 5 Department of Dermatology, Aarau, Switzerland; 6 Faculty of Medicine, University of Zürich (UZH), Zürich, Switzerland; 7 University Medical Center Utrecht & Princess Maxima Center, Utrecht, The Netherlands; 8 Comprehensive Cancer Center Munich of the Technical University Munich and the Ludwig Maximilians University, Munich, Germany; 9 Charité – Universitätsmedizin Berlin, corporate member of Freie Universität Berlin and Humboldt Universität zu Berlin, Department of Dermatology, Venereology and Allergology, Berlin, Germany; 10 Molecular and Pharmaco-Epidemiology Unit, European Institute of Oncology, IRCCS, Milano, Italy; 11 Department of Surgical Oncology, The University of Texas MD Anderson Cancer Center, Houston, TX USA; 12 Department of Dermatology, University Hospital Schleswig-Holstein (UKSH), Campus Kiel, Kiel, Germany; 13 Department of Dermatology, Medical University of Vienna, Vienna, Austria; 14 Sandra and Thomas Usher Chair in Melanoma Research, Melanoma and Skin Cancer Program, University of Pittsburgh & UPMC Hillman Cancer Center, Pittsburgh, PA USA; 15 Université Paris Cite, AP-HP Dermatooncology and CIC, Cancer institute APHP.nord Paris cité, INSERM U1342, Saint Louis Hospital, Paris, France; 16 Melanoma Institute Australia, The University of Sydney, and Royal North Shore and Mater Hospitals, Sydney, Faculty of Medicine & Health, The University of Sydney, Sydney, Australia; 17 University of Manchester and Christie NHS Foundation, Manchester, UK; 18 Immunotherapy and Drug Development Center, UPMC Hillman Cancer Center and University of Pittsburgh, Pittsburgh, PA USA; 19 Skin Cancer Center at the University Cancer Center Dresden and National Center for Tumor Diseases Dresden, Dresden, Germany; 20 Department of Dermatology, Faculty of Medicine and University Hospital Carl Gustav Carus, Technische Universität Dresden, Dresden, Germany; 21 Mount-Vernon Cancer Centre, Northwood, United Kingdom; 22 Department of Medicine, Memorial Sloan Kettering Cancer Center, New York City, and Weill Cornell Medical College, New York City, NY USA; 23 Department of Medical Oncology, Gustave Roussy and Paris Saclay University, Villejuif, France; 24 Department of Dermatology, University Hospital Essen, Essen, & German Cancer Consortium, Heidelberg Partner Site Essen, Essen, Germany; 25 Population Health Department, QIMR Berghofer Medical Research Institute, Brisbane, QLD Australia; 26 Institute for Clinical Epidemiology and Applied Biometrics, University of Tuebingen, Tuebingen, Germany

**Keywords:** Cancer, Oncology

## Abstract

A false-negative diagnosis of cancer can lead to a delay in effective treatment and a poorer prognosis. Here, we use the example of cutaneous melanoma to examine how many years of life are lost after a false-negative diagnosis compared to a primarily correct diagnosis. From 1996 to 2015, 9,063 patients are prospectively registered in the German Central Malignant Melanoma Registry and followed up until December 2023. A false-negative diagnosis is found in 206 (2.3%) patients. The median time to correct diagnosis is 24.0 months. The 10-year recurrence-free survival probabilities are 32.9% for false-negative diagnoses and 76.2% for correct diagnoses (p < 0.001). The 10-year melanoma-specific survival probabilities are 62.1% versus 85.0% (p < 0.001). On average, each person with an initial false-negative diagnosis loses 8.2 years of life compared to people with a correct diagnosis. This high number of years of life lost raises the question of whether similar results also apply to other types of cancer.

## Abstract (web summary)

A false-negative diagnosis (FND) is associated with a delayed true positive diagnosis and treatment. Here the authors show the accuracy of melanoma diagnosis is high with only 2.3% of the patients experiencing FNDs and an expected 7-8 years of life lost.

## Introduction

Early detection of primary melanoma is associated with more favorable prognosis and improved prognostic factors: lack of invasiveness when diagnosed as melanoma in situ, low tumor thickness in early invasive melanomas, as well as the absence of ulceration. A delay in diagnosis is often caused by the patient waiting too long before seeing a doctor^1^. Another cause of delayed diagnosis could be a primary misdiagnosis^2^.

There are three main categories of misdiagnosis in medicine: (1) false positive: misdiagnosis of a disease that is not actually present. (2) false-negative: failure to diagnose a disease that is present. (3) Equivocal results: inconclusive interpretation without definitive diagnosis^3^. A false positive diagnosis worries the patient, but has no negative consequences for the course of the disease. The false positive diagnosis is usually not recognized, even later in the clinical pathway. A false-negative diagnosis (FND) is associated with a delayed true positive diagnosis and delayed treatment and usually has a negative effect on the course of the disease.

There are two types of FND in melanoma^4^: In the case of clinical FND, the lesion is not biopsied, never undergoes histological examination and is therefore considered benign. The correct diagnosis is usually established later as a result of the patients noticing some further change in the lesion and consulting a doctor again, leading to a biopsy and the correct diagnosis. In contrast, histopathological FND is only recognized retrospectively when a recurrence or metastasis occurs. This study focuses on both types of FND in melanoma and their effects on the course of the disease. FND has been reported to be more frequent in acral melanomas, particularly on the foot^5–7^. FND has also been found to be more common in ano-rectal melanomas^8^.

Here we report on the results of a prospective study conducted by the German Central Malignant Melanoma Registry (CMMR) since 1996 to analyze the frequency of FND in primary melanomas and its impact on patient prognosis. The analysis distinguishes between clinical and histopathological FND. The number of years of life lost (YLL) in patients with FND is determined by comparison with patients with a true-positive diagnosis (TPD). We analyze the total number of YLL in patients with FND as well as its variation by sex, age, localization, and other factors. The impact of FND is also characterized by using survival curves for recurrence-free survival and melanoma-specific survival. To our knowledge, there are no studies to date on the number of YLL after FND in melanoma, nor are there any corresponding analyses for other types of solid cancers.

## Results

### Patient characteristics and prognostic factors

The study cohort consisted of 9,063 patients (male: *n* = 4591, female: *n* = 4472), including 206 (2.3%) classified with an initial clinical or histopathological FND (male: *n* = 87, female: *n* = 119), and 8857 TPD patients (male: *n* = 4504, female: *n* = 4353). Among patients with FND, women predominated at 57.8% compared to patients with TPD at 49.1% (χ² (1) = 5.98, *p* = 0.014).

The median age at correct positive diagnosis was 58.2 years in FND patients and 57.7 years in TPD patients. The median tumor thickness was 2.11 mm in the FND collective (*n* = 150) and 0.89 mm in the TPD collective (*n* = 8731), (U = 366 935, *z *= − 9.25, *p* < 0.001). Ulceration was present in 22.3% (*n* = 46) of primary lesions in FND patients and in 15.6% (*n* = 1384) in TPD patients (χ² (1) = 6.81, *p* = 0.009). 10.3% of patients with TPD and 34.5% with FND presented with locoregional or distant metastasis (χ² (4) = 242.3, *p* < 0.001). The median follow-up time was 81.5 months [32.8, 147.3] for the FND group, and 91.0 months [44.0, 123.0] for the TPD group. (Table 1, Table 2).

**Table 1:** Description of clinical and histopathological characteristics of falsely negative diagnosed (FND) vs. truly positive diagnosed (TPD) melanoma patients recorded by the Center for Dermatooncology, University of Tuebingen, Germany (1996–2015, follow-up until 2023)

	False-negative diagnosis (*n* = 206)	True-positive diagnosis (*n* = 8857)	*p*-value
**Age**			
Median [yrs] (IQR) at FND	54.8 (42.7;66.1)	–	–
Median [yrs] (IQR) at correct diag.	58.2 (46.4;68.0)	57.7 (44.3;69.6)	0.881
Median [yrs] (IQR) at death of MM	59.8 (50.2;71.1)	67.2 (54.6; 77.4)	< 0.001
**Gender**			
Male	87 (42.2%)	4504 (50.9%)	0.014
Female	119 (57.8%)	4353 (49.1%)	–
Missing	0 (0%)	0 (0%)	–
**Stage at diagnosis**			
I	60 (29.1%)	6139 (69.3%)	< 0.001
II	54 (26.2%)	1635 (18.5%)	–
III	61 (29.6%)	850 (9.6%)	–
IV	10 (4.9%)	64 (0.7%)	–
Missing	21 (10.2%)	169 (1.9%)	–
**Tumor thickness**			
Median [mm] (IQR)	2.11 (1.1, 4.0)	0.89 (0.45, 1.9)	< 0.001
< 1.00 mm	34 (16.5%)	4882 (55.1%)	–
1.01- 2.00 mm	38 (18.4%)	1873 (21.2%)	–
2.01- 4.00 mm	41 (19.9%)	1268 (14.3%)	–
> 4.00 mm	37 (18.0%)	708 (8.0%)	–
Missing	56 (27.2%)	126 (1.4%)	–
**Level of invasion**			
II	6 (2.9%)	1792 (20.2%)	< 0.001
III	26 (12.6%)	2415 (27.3%)	–
IV	82 (39.8%)	3554 (40.1%)	–
V	19 (9,3%)	280 (3.2%)	–
Missing	73 (35.4%)	816 (9.2%)	–
**Histological subtype**			
SSM	47 (22.8%)	5586 (63.0%)	< 0.001
NM	35 (17.0%)	1153 (13.0%)	–
LMM	18 (8.7%)	806 (9.1%)	–
ALM	49 (23.8%)	352 (4.0%)	–
Others	26 (12.6%)	599 (6.8%)	–
Missing	31 (15.1%)	361 (4.1%)	–
**Ulceration**			
No/not reported	160 (77.7%)	7473 (84.4%)	0.009
Yes	46 (22.3%)	1384 (15.6%)	–
Missing	0 (0%)	0 (0%)	–
**Localization**			
Head, scalp, and neck	42 (20.4%)	1474 (16.6%)	< 0.001
Anterior trunk	7 (3.4%)	1043 (11.8%)	–
Posterior trunk	28 (13.6%)	2573 (29.1%)	–
Upper extremities	20 (9.7%)	1281 (14.5%)	–
Lower extremities	30 (14.6%)	1947 (22.0%)	–
Hand/Foot (Acral)	79 (38.3%)	539 (6.1%)	–
Missing	0 (0%)	0 (0%)	–

*IQR* interquartile range, *SSM* superficial spreading melanoma, *NM* nodular melanoma, *LMM* lentigo maligna melanoma, *ALM* acral lentiginous melanoma.

**Table 2:** Follow-up and FND-specific data of FND vs. TPD melanoma patients recorded by the Center for Dermatooncology, University of Tuebingen, Germany (1996–2015, follow-up until 2023)

	False-negative diagnosis (*n* = 206)	True-positive diagnosis (*n* = 8857)	*p*-value
**Follow-up time**^a^			
Median [months] (IQR)	81.5 (32.8,147.3)	91.0 (44.0,123.0)	0.630
**Death from melanoma**	82 (39.8%)	1106 (12.5%)	< 0.001
**Recurrences during Follow-up**			
Total	142 (68.9%)	1820 (20.5%)	< 0.001
Satellite/in-transit metastases	64 (31.1%)	783 (8.8%)	< 0.001
Regional lymph node metastases	71 (34.5%)	938 (10.6%)	< 0.001
Distant metastases	89 (43.2%)	1332 (15.0%)	< 0.001
**Type of FND**			
Clinical	106 (51.1%)	–	–
Histopathological	100 (48.5%)	–	–
**Time delay (months)**			
Median [IQR]			
Total	24.0 [10.0; 42.3]	**–**	–
Clinical	19.0 [7.8; 25.3]	–	–
Histopathological	29.0 [13.0; 54.8]	–	–

*P* values were calculated using Pearson's chi-square test for categorical variables and Mann-Whitney *U* test for continuous variables, all statistical tests were performed two-sided. ^a^Calculated from date of first diagnosis (TPD) until death or censoring.

Incomplete data, particularly in the group of FND patients, were found for stage at initial diagnosis (*n* = 21, 10.2%), tumor thickness (*n* = 56, 27.2%), level of invasion (*n* = 73, 35.4%), and for histological subtype (*n* = 31, 15.1%). (Table 1).

### Characteristics of false-negatively diagnosed melanomas

In 206 FND patients, clinical FND was present in 106 (51.5%) patients and histopathological FND was found in 100 patients (48.5%). Clinical FND were strongly associated with anatomic location of the melanoma; 60 of 106 occurred on acral sites (56.6%) compared to 539 in 8,857 TPD patients (6.1%). Most frequently, these were subungual melanomas (26 of 60; 43.3%), followed by plantar melanomas (14 of 60; 23.3%).

The median delay until later TPD for all FND patients (*n* = 206) was 24.0 [10.0; 42.3] months. For patients with a clinical FND (*n* = 106) the median delay accounted for 19.0 [7.8; 25.3] months, and for histopathological FND (*n* = 100) the delay was 29.0 [13.0; 54.8] months (Table 2).

### Survival analyses

Death from melanoma occurred in 82 (39.8%) of FND patients, compared to 1106 (12.5%) in the TPD group (χ² (1) = 131.9, *p* < 0.001) (Table 2).

**Fig. 1:**
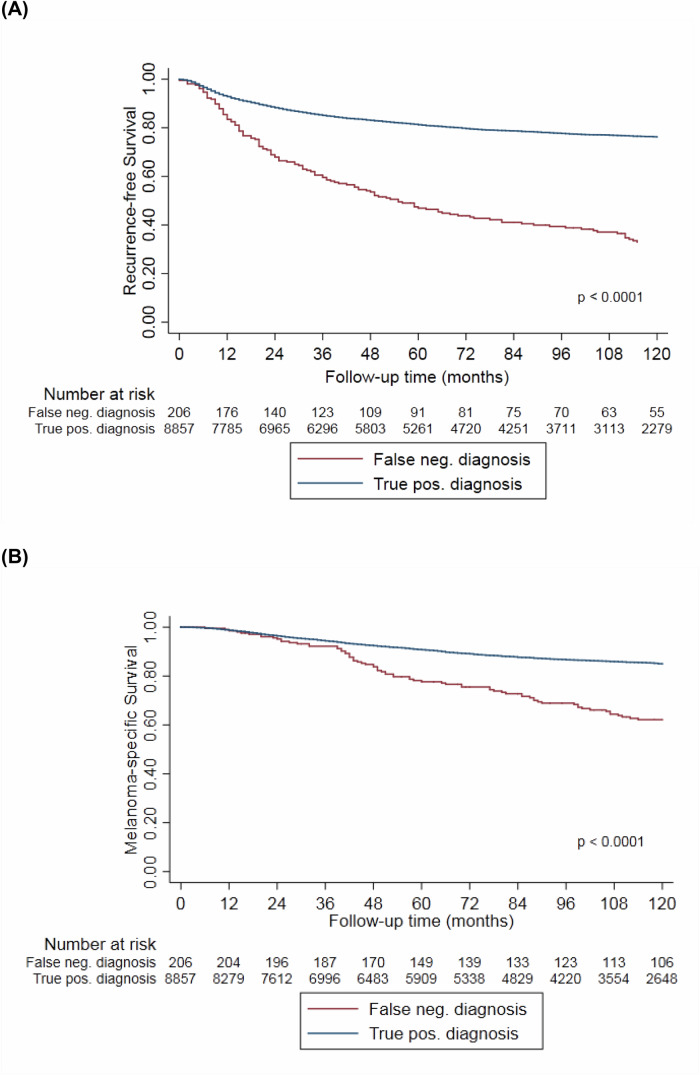
Survival probabilities and patient number at risk, according to false-negative and true-positive diagnosis of melanoma. **A** Recurrence-free Survival, χ² (1) = 237.74, *p* < 0.001, log-rank test), (**B**) Melanoma-specific Survival, χ² (1) = 51.04, *p* < 0.001, log-rank test). All tests were performed two-sided.

The 5- and 10-year recurrence-free survival probabilities for FND (*n* = 206) vs. TPD (*n* = 8857) were 47.5% (95% CI: 40.6, 54.4) vs. 81.4% (95%CI: 80.6, 82.2, χ² (1) = 237.74, *p* < 0.001) and 32.9% (95%CI: 26.2, 39.6) vs. 76.2% (95%CI: 75.2, 77.2, χ² (1) = 237.74, *p* < 0.001). The 5- and 10-year melanoma-specific survival probabilities for FND (*n* = 206) vs. TPD (*n* = 8857) were 78.2% (95% CI: 72.5, 83.9) vs. 90.8% (95% CI: 90.2, 91.4, χ² (1) = 51.04, *p* < 0.001) and 62.1% (95%CI: 55.2, 69.0) vs. 85.0% (95%CI: 84.0, 86.0, χ² (1) = 51.04, *p* < 0.001). (Table 3 and Fig. 1)

**Table 3:** Survival probabilities for recurrence-free and melanoma-specific survival in false-negative and in true-positive diagnoses

	False-negative diagnosis (*n* = 206)	True-positive diagnosis (*n* = 8857)	*p*-value
**Recurrence-free Survival**			
5-yr. rate (95% CI*)	47.5% (40.6; 54.4)	81.4% (80.6; 82.2)	< 0.001
10-yr. rate (95% CI)	32.9% (26.2; 39.6)	76.2% (75.2; 77.2)	–
Median [IQR]	55.0 months [20.0; -]	not reached	–
**Melanoma-specific Survival**			
5-yr. rate (95% CI)	78.2% (72.5; 83.9)	90.8% (90.2; 91.4)	< 0.001
10-yr. rate (95% CI)	62.1% (55.2; 69.0)	85.0% (84.0; 86.0)	–
Median [IQR]	not reached	not reached	–

Abbreviation: *CI Confidence Interval. *P*-values for survival analyses were calculated using log-rank tests, all statistical tests were performed two-sided.

Further Kaplan-Meier analyses of MSS in the total sample (FND and TPD, *n* = 9063) showed that age at first diagnosis (*n* = 9063), AJCC stage (*n* = 8873), tumor thickness (*n* = 8881), Clark level of invasion (*n* = 8174), histopathological subtype (*n* = 8671), anatomic location of primary tumor (*n* = 9063), localization on acral sites (*n* = 9063), all *p* < 0.001, gender (*n* = 9063, *p* = 0.014) and ulceration (*n* = 9063, *p* = 0.009) were significant prognostic factors in univariate analysis, based on the results of the log-rank test (data not shown).

### Independent prognostic factors in multivariate Cox proportional hazard analysis

To assess the independent effect of FND on MSS, a multivariate Cox proportional hazard analysis (*n* = 9063), adjusted for gender (male: *n* = 4591; female: *n* = 4472), acral melanoma (yes: *n* = 618; no: *n* = 8445) and age at first diagnosis (*n* = 9063, as a continuous variable) and an interaction term (*n* = 9083) between FND and acral melanoma was performed (Table 4).

**Table 4:** Prognostic factors of melanoma-specific survival in patients with cutaneous melanoma (*n* = 9063) with true-positive and false-negative diagnosis. Results of the multivariate Cox proportional hazard analysis

Melanoma-specific Survival			
Prognostic factor	HR	95% CI	*p*-value
**Gender**	–	–	< 0.001
Female	1	–	–
Male	1.60	1.43; 1.81	–
**Age (1**^**st**^ **diagnosis)**	1.02	1.02; 1.03	< 0.001
**FND**	–	–	< 0.001
No	1	–	–
Yes	2.14	1.56; 2.94	–
**Acral MM**	–	–	< 0.001
No	1	2.00; 2.84	–
Yes	2.38	–	–
FND*Acral MM Interaction	0.76	0.48; 1.21	0.251

Abbreviation: *HR* Hazard Ratio, *CI* Confidence Interval. Reported *p*-values are from the Wald test within the Cox regression model, all statistical tests were performed two-sided.

All factors included in the model were identified as significant prognostic factors for MSS (*p* < 0.05; Wald Test). No significant interaction was found between FND and acral MM (Wald-χ² (1) = − 0.274, *p* = 0.251, HR = 0.76). Both FND (Wald-χ² (1) = 22.09, *p* < 0.001, HR = 2.14) and acral localization (Wald-χ² (1) = 93.37, *p* < 0.001, HR = 2.38) remained significant independent prognostic factors for MSS Table 4.

### Numbers of years of life lost

The average number of YLL due to FND of melanoma (*n* = 206) was 8.2 (method 1) and 6.9 years (method 2), respectively, compared to patients with a TPD (*n* = 8857) of melanoma (Table 5).

**Table 5:** Estimates of years of life lost in subjects with false-negative diagnosis compared to those with true-positive diagnosis of melanoma

	Average years of life lost (± SD)			
	False-negative Diagnosis (*n *= 206)	True-positive Diagnosis (*n* = 8857)	Difference in average years of life lost	*p*-value
Method 1*	7.56 ( ± 16.3)	− 0.62** (± 9.0)	8.18	< 0.001
Method 2*	7.89 ( ± 15.8)	1.07 ( ± 8.8)	6.92	< 0.001

Abbreviation: *SD* Standard Deviation. *P*-values were calculated using the non-parametric Mann-Whitney U-test, all statistical tests were performed two-sided.

*Method 1: YLL were calculated as the difference between life expectancy (life tables for year of birth, sex, and age at diagnosis) and observed lifetime in patients with melanoma-specific death. For censored patients, life expectancy was estimated using life tables after 15 years of follow-up. Life expectancy of censored cases with FND was corrected for the interval between censoring and 15 years using Kaplan-Meier estimates. **Method 2**: YLL were calculated as the difference from life expectancy (life tables for year of birth, gender and age at diagnosis) and observed life time in patients with melanoma specific death or for censored patients the life expectancy was estimated from life tables after censoring. (See the formulas for the calculation in the supplementary material.)

**The **negative value** indicates a slightly better survival of the melanoma patients than of the general population. This is known for low-risk melanoma patients who have been cured by surgery and have a higher life expectancy due to their high education, high income and high socio-economic status (Education, income and socio-economic status are not reported in this database.).

A multivariate linear regression model, adjusted for age, gender and localization, was fit, to evaluate whether the type of FND (clinical vs. histopathological) and time delay between first FND and TPD ( ≤ 60 months > 60 months) are independent significant prognostic factors for the number of YLL in a cohort of FND patients (*n* = 206).

Interestingly, neither type of FND (Wald-χ² (1) = 0.710,* p* = 0.399) nor time delay (Wald-χ² (1) = 0.717, *p* = 0.397) could be identified as significant predictors for the number of YLL. In contrast, age (Wald-χ² (2) = 25.86, *p* < 0.001), gender (Wald-χ² (1) = 5.51, *p* = 0. 019), and localization (Wald-χ² (1) = 5.63, *p* = 0.018) were found to be significant predictors. The number of YLL was highest in the age group ≤ 45 yrs (*n* = 61, YLL = 17.6 yrs.) and decreased significantly from 9.0 yrs. in the age group 46–65 yrs (*n* = 92) to 2.81 yrs. in patients older than 65 yrs (*n* = 53). Additionally, male patients (*n* = 87) lost an average of 12.3 years of life, compared to 7.27 years in female patients (*n* = 119), (Table 6).

**Table 6:** Average years of life lost by age at first diagnosis, gender, localization (acral vs. non-acral), type of FND, and time delay for patients with FND of melanoma (*n* = 206) *

Prognostic factors	Adjusted Mean years of life lost (95% CI)	*p*-value
**Age at 1**^**st**^ **diagnosis**		
≤ 45 yrs. (*n* = 61)	17.6 (13.2, 22.0)	< 0.001
46–65 yrs. (*n* = 92)	9.00 (5.1, 12.9)	–
> 65 yrs. (*n* = 53)	2.81 (− 2.1, 7.7)	–
**Gender**		
Male (*n* = 87)	12.3 (8.2, 16.5)	0.019
Female (*n* = 119)	7.27 (3.8, 10.7)	–
**Localization**		
Acral (*n* = 79)	12.6 (8.3, 16.8)	0.018
Non-acral (*n* = 127)	7.03 (3.5, 10.5)	–
**Type of FND**		
Histopathological (*n* = 100)	10.8 (6.9, 14.6)	0.399
Clinical (*n* = 106)	8.83 (4.9, 12.8)	–
**Time delay**		
≤ 60 mo. (*n* = 179)	8.45 (6.1, 10.8)	0.397
> 60 mo. (*n* = 27)	11.1 (5.3, 17.0)	–

^*^Estimates are based on method 2,

Abbreviation: *CI Confidence Interval. Reported *p*-values are from the Wald Chi-Square test within the generalized linear model; all statistical tests were performed two-sided.

In FND patients who died from melanoma (*n* = 82), the average YLL was about 22.4 years higher than in patients who died from other causes (*n* = 28).

### Type of false-negative diagnoses and specialty of the diagnosing physicians

Only the presence of FND and TPD were prospectively documented, but not the type of FND and the specialties of the diagnosing physicians. For a total of 164 patients, further data on the type of FND and the specialties of the diagnosing physicians were available in the hospital’s patient files and could be collected retrospectively.

The clinical FNDs (*n *= 73) were diagnosed in 44% by general practitioners, in 41% by dermatologists, and in 15% by surgeons and other physicians. The most common clinical FND (*n* = 56) were mycoses or verrucae vulgares (Supplementary Table S1). The clinical FND were found in 54% of acral localizations on the hands and feet. Half of these were in subungual localization, and the most common FND was onychomycosis. (Supplementary Table S2).

The histopathologic FNDs (*n* = 73) were diagnosed in 70% by general pathologists and in 30% by dermato-histopathologists. Benign melanocytic nevi and variants were diagnosed 77%.

The remaining histopathological FND were tumors of other tissue origin. (Supplementary Table S3)

### False-negative diagnoses of acral and non-acral melanomas

False-negative diagnoses of acral melanoma (*n* = 79) occurred more frequently in women and in older age groups than non-acral melanoma (*n* = 127). They were associated with greater tumor thickness and more ulceration and had a particularly unfavorable prognosis (Supplementary Tables S4 and S5). The number YLL in patients with acral melanomas (YLL = 12.6 years) was significantly higher compared to those with non-acral melanomas (YLL = 7.03 years).

## Discussion

More than 9,000 patients with primary cutaneous melanoma were enrolled in this study over a period of 20 years with an additional follow-up of 8 years. Patients who had a follow-up time of at least 3 months were included in this study. Our intention was to distinguish between patients with and without follow-up, as the shortest time interval according to German guidelines is three months. The probability of dying from melanoma in the first three months after the initial diagnosis is very low. This approach was also used in other CMMR studies^9–11^. Of the 9063 patients 2.3% had initially an FND. The initial FND was associated with substantial loss of years of life. Patients with a FND lost on average 8 years of life (according to method 1) or 7 years of life (according to method 2) compared to patients with an initial TPD. In the FND group, 39.8% died of melanoma compared to 12.5% in the TPD group.

The percentage of FND of melanoma is lower than reported for several case series in the literature. This may be due to the fact that the patients in our study were seen in a large referral center specializing in melanoma. Clinical FND were made in a rather high percentage by general physicians and were most frequent in acral melanomas, a phenomenon which has been previously reported in the literature^12–15^. FND were only recorded if a TPD was subsequently made. FND without a subsequent TPD could not be detected. In clinical FND, it cannot be completely ruled out that the lesion was still benign at the time of the initial diagnosis and only later became malignant. However, this probability is very low and cannot be evaluated more precisely. Two-thirds of all primary melanomas arise de novo and are not developed from pre-existing nevi; these are malignant from the very beginning^16–18^.

Histopathologic FND often involved the misdiagnosis of a benign melanocytic nevus and occurred most frequently when diagnosed by general pathologists, whereas the majority of melanoma cases in the total population are diagnosed by dermatopathologists^19,20^. Histopathologic FNDs could only be detected if tumor recurrence subsequently occurred. All histopathological FNDs without subsequent tumor recurrence are not included in the analysis. The YLL expectancy would possibly be less if these cases were detectable. Histopathologic misdiagnosis may lead to a higher frequency of recurrence due to the omission of a safety margin excision and the lack of indication for a sentinel lymph node biopsy, which is only performed in cases of melanoma diagnosis.

Histopathologic FND of melanoma plays an exceptional role: A FND of melanoma is the single most common reason for filing a malpractice claim against a pathologist in the US^21^. Clinical FND were mostly revised due to clinical changes of the lesion, to further growth or due to the development of loco-regional or distant metastasis^22,23^.

The FND was associated with a considerable delay of correct positive diagnosis and subsequent adequate treatment. On average, the delay was 24 months. For clinical FND, it was 19 months and for histopathological FND, 29 months (Table 2). During this time, the primary tumors continued to grow and/or metastasized. Accordingly, the patients with FND were treated at higher TNM stages, with a greater tumor thickness and a higher frequency of ulcerations. The 19-month delay in diagnosis for clinical FND is associated with an increase in median tumor thickness from 0.89 mm (TPD) to 2.11 mm (FND) (Table 1), i.e., an increase of 1.2 mm. The growth rate of the primary melanoma can therefore be considered slow^24–27^.

Medical FND causes a delay in diagnosis in a small percentage of patients. In comparison, the delay in diagnosis by patients themselves is considered to be more significant. Approximately one-third of all patients wait more than a year to see a doctor after observing the first signs of melanoma^23,28,29^.

Misdiagnosis was more common in patients with FND localized in the acral area (38.3% of all FND vs. 6.1% of all TPD). Misdiagnosis of acral melanoma is a particular subtype of FND that is associated with greater tumor thickness, a much higher rate of ulceration, and a significantly less favorable prognosis than for FND of non-acral melanoma. This applies especially to subungual melanomas, which are often wrongly categorized as tinea, trauma or even warts, especially by GPs but also by dermatologists. Another factor contributing to the poorer outcome of acral melanoma is the lower response rates to immunotherapy and targeted therapies in the metastatic stages.

The high number of YLL of patients with FND compared to patients with initial TPD was surprising. Using two different mathematical models, there was a loss of approximately 7-8 years of life in patients with FND. YLL was significantly higher in younger people than in older people and higher in men than in women.

Until now, YLL have been calculated only for patients who actually died of melanoma, resulting in a figure of 17 YLL^30^. In this study, we developed a mathematical model that takes into account the less favorable course of the censored cases in the Kaplan-Meier curve to calculate the resulting YLL. The calculations were based on life expectancy values, which are given in official life tables for each year of birth, the gender and age of each individual.

Calculations of the expected years of life lost through FND of cancer have to be performed with the highest achievable accuracy. It is likely that such data will be used in malpractice suits. In order to achieve the most reliable results possible, we chose a long follow-up time for our patient cohorts. No such data was available in the past. The present data seems to be fairly representative for melanoma with FND.

The present analysis has limitations. The percentage of patients with FND may have been underreported, particularly among patients who had an early-stage melanoma that was never diagnosed with the biopsy performed, and was at low risk for recurrence that might have triggered an FND. This may also contribute to an overestimation of YLL. The type of FND and the specialty of the diagnosing physician were not prospectively documented. Unfortunately, we did not have data on socioeconomic status, so we were unable to analyze whether FND is associated with poverty. The endpoint of melanoma-specific survival depends on the quality of the data on deaths and causes of death. Despite active strategies to collect this data, we are unlikely to achieve the same quality for this endpoint as in prospective randomized studies. Another limitation is that this study has been performed in a single center. This, however, gave us the opportunity to recheck the files of every single patient with FND.

The strength of this study lies in the further elaboration of the calculation of YLL. Two mathematical models have been developed to estimate expected YLL for patients being censored during follow-up. Another strength of the study is the solid documentation and the very long follow-up time of all patients.

In conclusion, in our study population, the accuracy of melanoma diagnosis is quite high, and only in 2.3% of patients was an initial FND found. However, an average of expected 7-8 years of life lost was calculated for these patients. Methods of quality assurance should be considered in order to minimize FND with substantial YLL. It would be interesting to analyze whether similarly high losses of life years are also found in primary false-negative diagnoses of other cancers.

## Methods

### Ethical statement

This study was approved by the Ethics Committee at the Medical Faculty of Eberhard Karls University and at Tübingen University Hospital (Project ID number: 574/2025BO2). The Ethics Committee is registered with the federal authorities. All procedures performed were in accordance with the ICH-GCP guidelines, the Declaration of Helsinki in its currently valid version, and the applicable legal provisions. Potential conflicts of interest of the authors are listed at the Competing Interests Statement. The authors declare no other competing interests.

### Patients

The Central Malignant Melanoma Registry (CMMR) of the German Dermatological Society was established in 1983, and more than 140,000 cutaneous melanomas were registered until the end of 2023^31,32^. The CMMR is a hospital-based registry. Approximately 60 departments in Germany have documented their patients on a voluntary basis without remuneration. Demographic data, tumor data, basic data on treatment, follow-up and the course of the disease were recorded.

This study of the German CMMR is based on a prospective documentation of FND and TPD at the University Department of Dermatology, Tuebingen, Germany. Trained study personnel reviewed the case notes of all patients attending the department with newly incident melanoma to determine whether the same lesion had been diagnosed previously as a benign lesion; the date of the first such examination was taken as the FND date and used for survival calculations. An active approach to recording death data and causes of death for the CMMR has been established at the University Hospital of Tübingen. If follow-up was discontinued, the family, the treating physicians, or even the residents’ registration offices were contacted for information. All patients provided a written informed consent for their data documentation and follow-up documentation in the CMMR. Patients were enrolled from January 1996 to December 2015, and the follow-up was continued until December 2023. Only patients with primary invasive melanoma and with a minimum follow-up time of three months after diagnosis were included in the study. Patients with metastatic melanoma of unknown primary, with mucosal or ocular melanoma, were excluded.

### Histopathological diagnoses

The histopathological diagnoses were made by trained dermatopathologists (JB, SF, CG, GM, and others) who participated in regular continuing education courses (German Society of Dermatological Histology). Melanoma diagnoses were made jointly by at least two examiners. In cases of suspected misdiagnosis by external examiners, the specimens were re-requested and re-evaluated, which is standard procedure in Germany. Immunohistological staining was generally performed to confirm the diagnosis. The sections were not scanned.

### False-negative diagnosis of primary melanoma

When documenting cases from the patient file in the CMMR data set, the medical documentation assistant decided whether a primary FND was present based on the anamnestic information and documented it prospectively. This was only answered with “yes” if the information clearly indicated this. Furthermore, the date of the primary FND was also documented prospectively. Additional data on the FND, such as the exact type of diagnosis and the specialty of the diagnosing physician, were later collected retrospectively from the patient files. FND were classified either as clinical or histopathological misdiagnoses. Clinical FND were mainly made by general physicians and dermatologists, when they interpreted a skin lesion as benign and observed the lesion without performing a biopsy. Histopathological FND occurred when a biopsy was initially misdiagnosed as benign but was later reclassified as melanoma, usually after recurrence and a second histopathologic report.

### Outcome

The primary outcome of this study was the number of YLL of patients with an FND compared to patients with an initial TPD, calculated from the date of first FND or TPD.

The secondary outcomes were recurrence-free survival (RFS) and melanoma-specific survival (MSS). Survival was defined as the time between date of first FND or TPD and the date of first recurrence or the date of death from melanoma, the follow-up of patients still alive was censored at the last date known to be alive.

### Calculation of years of life lost

For each patient dying from melanoma, the individual YLL was calculated by subtracting the actual survival since FND or TPD from the remaining life expectancy at the time of first diagnosis, based on established Generation Life Tables for Germany. Life expectancy was obtained as a function of year of birth, age at first diagnosis and gender. For patients being censored during follow-up, years of life lived after diagnosis are calculated as the sum of the actual survival time until censoring and the expected years of life after censoring. The YLL due to FND were calculated by the difference of YLL between FND and TPD patients. To estimate the expected years of life after censoring, two different methods have been applied (Supplementary Fig. S1):

**Method 1** is based on the assumption that melanoma patients have a less favorable prognosis than the normal population. As the broad majority of melanoma related deaths occur until year 15 after initial diagnosis, a cut-off point of 15 years of observation was chosen. All patients still alive at year 15 were censored, and their observed survival(∆t_1_obs_)was set to 15 years. For these patients, expected years of life after cut-off (∆t_3_exp_)are directly obtained from demographic life expectancy tables for year of birth, age at cut-off and gender. For patients being censored before year 15, the expected survival after censoring is calculated by the sum of two estimates (∆t_2_exp_ and ∆t_3_exp_)^.^

Life expectancy (∆t_2_exp_) from time point of censoring until cut-off is based on survival probabilities, obtained from the Kaplan Meier curve. For each patient, the total time from censoring until cut-off is divided into multiple time intervals defined by subsequently occurring deaths or censoring. Each interval has to be adjusted for the probability to survive the time period from start until end of the interval, given to have survived until the beginning of the interval. The total life expectancy (∆t_2_exp_) is calculated by the sum of all intervals.

The expected years of life after year 15 (∆t_3_exp_) for patients being censored before year 15 refers to the demographic life expectancy at the age of the cut-off point, also adjusted for conditional probabilities (= probability of surviving the time interval from censoring until cut-off, given to have survived until the time point of censoring).

The formula for the calculation of years of life lost (Y_1_) is as follows:

1 $${Y}_{1}=\frac{{\sum }_{n=1}^{{cN}}\left({C}_{{ex}_{diag}}\right)-{\sum }_{n=1}^{{cN}}\left({\varDelta} {t}_{{1}_{obs}}+{\varDelta} {t}_{2\_{exp }}+{\varDelta} {t}_{3\_{exp }}\right)}{{cN}} \\ -\frac{{\sum }_{n=1}^{{fN}}\left({f}_{{ex}\_{diag}}\right)-{\sum }_{n=1}^{{fN}}\left({\varDelta} {t}_{{1}\_{obs}}+{\varDelta} {t}_{2\_{exp }}+{\varDelta} {t}_{3\_{exp }}\right)}{{fN}}$$

with

2 $${\varDelta} {t}_{1\_{obs}}={x}_{{death}/{censoring}}-{x}_{{diagnosis}}$$

3 $${\varDelta} {t}_{2\_{exp}}=\\ {\sum }_{j={{{\rm{i}}}}}^{k-1}\left[\left({x}_{j+1}-{x}_{j}\right){x}_{p\left({x}_{j}\right)}^{\,p({x}_{j+1})}\right];{x}_{j}\,{is}\,{individually}\,{different},{x}_{k}\,{{is}}\,{{constant}}\,(={cut}-{off})$$

4 $$	\frac{p({x}_{j+1})}{p\left({x}_{j}\right)}=\\ 	{probability}\,{of}\,{surviving}\,{the}\,{time}\,{period}\,{x}_{j}\,{to}\,{x}_{j+1}\,{given}\,{to}\,{have}\,{survived}\,{until}\,{x}_{j}$$

5 $${\varDelta} {t}_{3\_{exp}}=\frac{p\left(x_{{cut}-{off}}\right)}{p\left({x}_{{censoring}}\right)}x\left({e}_{x{cut}-{off}}\right)$$

6 $$	\frac{p\left(x_{{cut}-{off}}\right)}{p\left({x}_{{censoring}}\right)}\\ 	={probability}\,{of}\,{surviving}\,{the}\,{time}\,{period}\,{x}_{{censoring}}\,{to}\,x_{{cut}-{off}},\\ 	{given}\,{to}\,{have}\,{surved}\,{until}\,{x}_{{censoring}}$$

7 $${e}_{x_{cut}-{off}}={life}\,{expectancy}\,{at}\,{cut}-{off}\,{point}\,{according}\,{to}\,{life}\,{table}$$

ce_x_diag_: expected survival time (according to life table) at time point of first diagnosis for the cohort with TPD

fe_x_diag_: expected survival time (according to life table) at the time point of the first false diagnosis for the cohort with FND

∆t_1_obs_ observed survival time at the time point of first diagnosis/FND until death or censoring

∆t_2_exp_ expected survival time (according to Kaplan Meier curve) at the time point of censoring until the end of observation (cut-off), set to zero for deceased cases

∆t_3_exp_ expected survival time (according to life table) at the end of observation, set to zero for deceased cases

cN: number of patients of the cohort with TPD fN: number of patients of the cohort with FND

**Method 2** is based on the assumption that patients have a normal life expectancy at the time point of censoring based on the established Generation Life Tables for Germany. The expected ^years of life after censoring (^∆t_2_exp_) are obtained from demographic life tables for year of birth, age at censoring and gender. No adjustment was applied.

The formula for the calculation of years of life lost (Y_2_) is as follows:

8 $${Y}_{2}=\frac{{\sum }_{n=1}^{{cN}}\left({C}_{{ex}_{diag}}\right)-{\sum }_{n=1}^{{cN}}\left({\varDelta} {t}_{{1}_{obs}}+{\varDelta} {t}_{2\_{exp }}\right)}{{cN}}\\ -\frac{{\sum }_{n=1}^{{fN}}\left({f}_{{ex}\_{diag}}\right)-{\sum }_{n=1}^{{fN}}\left({\varDelta} {t}_{1\_{obs}}+{\varDelta} {t}_{2\_{exp }}\right)}{{fN}}$$

with

9 $${\varDelta} {t}_{{1}\_{obs}}={x}_{{death}/{censoring}}-{x}_{{diagnosis}}$$

10 $${\varDelta} {t}_{{2}\_{exp}}={e}_{{x}\_{{censoring}}}$$

11 $${e}_{x\_{censoring}}={life}\,{expectancy}\,{at}\,{censoring}\,{according}\,{to}\,{life}\,{table}$$

ce_x_diag_: expected survival time (according to life table) at time point of first diagnosis for the cohort with TPD

fe_x_diag_: expected survival time (according to life table) at the time point of the first false diagnosis for the cohort with FND

∆t_1_obs_ observed survival time at the time point of first diagnosis/FND until death or censoring

∆t_2_exp_ expected survival time (according to life table) at the time point of censoring, set to zero for deceased cases

cN: number of patients of the cohort with TPD

fN: number of patients of the cohort with FND

### Impact of prognostic factors on the number of years of life lost in false-negative diagnosis

A multivariate generalized linear model was employed to evaluate the impact of various prognostic factors considered significant predictors for the number of YLL among patients with FND. The model was fit with the following covariates: age at first diagnosis (≤ 45 yrs., 46–65 yrs., > 65 yrs.), gender (male vs. female), localization (acral vs. non-acral), type of FND (histo-pathological vs. clinical), and time delay (≤ 60 months vs. > 60 months). Results of the linear regression model were described by means of YLL and 95% confidence intervals. The reported *p*-values are from the Wald Chi-Square test within the generalized linear model.

### Statistics & reproducibility

No statistical method was used to predetermine sample size. Instead, all patients in the time period from January 1996 until December 2015 with a diagnosis of primary melanoma were included into the study and classified as FND or TPD. Randomization was not applicable.

The study was unblinded.

### Statistical analysis

Quantitative clinical and histopathological data were expressed by mean value and standard deviation or median value and inter-quartile range. Categorical data were presented as absolute numbers and proportions. Statistical testing of group differences was performed using chi-square tests for categorical and t-tests or non-parametric Mann-Whitney U-Tests for numerical data, depending on the distribution of the variable. No correction for multiple testing was applied. All statistical tests were two-sided, with a *p*-value ≤ 0.05 considered statistically significant. Statistical calculations were performed with IBM SPSS Statistics Version 28.0 (IBM SPSS, Chicago, IL) and STATA Version 18 statistical software (Stata Corp LLC, College Station, TX).

### Survival Analysis

The Kaplan-Meier method was used to estimate RFS and MSS, and the differences in survival were assessed by means of the log-rank test. Estimated 5- and 10-year survival probabilities were expressed as percentages with 95% confidence intervals.

A multivariate Cox proportional hazard analysis was used to establish the independent effect of FND on MSS, adjusted for potential confounders (gender, acral localization and age at first FND or TPD) and an interaction term between FND and acral localization. No adjustment was done for factors such as AJCC stage, tumor thickness and ulceration, as these factors are assumed to be on the causal pathway between exposure (FND) and outcome (MSS).

The assumption of proportional hazards was evaluated by visual inspection and testing of Schoenfeld residuals. Within a period of 120 months, the standard follow-up time for melanoma in Germany, no violation of the proportional hazards’ assumption was found (global test: *p* = 0.85). Results of the Cox proportional hazard analysis were described by Hazard Ratios (HRs) and 95% confidence intervals (CIs). The reported *p*-values are from the Wald test within the Cox model.

### Reporting summary

Further information on research design is available in the Nature Portfolio Reporting Summary linked to this article.

## Supplementary information

Supplementary Information

Reporting Summary

Transparent Peer Review file

## Data Availability

The data supporting the findings of this study are publicly available in the Zenodo repository at 10.5281/zenodo.20311307. Processed data used for the analyses, figures and tables are additionally available on GitHub (https://github.com/ukeim/FND_Study). The source data underlying the main and Supplementary Figs. and tables have been deposited in GitHub/Zenodo. Additional data are available from the corresponding author upon request. Restrictions apply to the availability of these data due to ethical and privacy considerations.

## Code availability

All codes used for data processing, statistical analyses, and figure generation is publicly available on GitHub (https://github.com/ukeim/FND_Study). A permanent archived version of the code is available on Zenodo at 10.5281/zenodo.20311307.
