# Supplementary material for: Years of life lost in patients with a false-negative diagnosis of primary melanoma. A prospective study of the German Central Malignant Melanoma Registry involving 9063 patients over 28 years
Source: Nat Commun. 2026 Jul 2;17:5790. doi: 10.1038/s41467-026-74443-9 (PMC13328647; doi:10.1038/s41467-026-74443-9)
Supplement: Supplementary file 1 — Supplementary Information [file 41467_2026_74443_MOESM1_ESM.pdf]

Supplementary Material

Table S1: Type and frequency of clinical false-negative diagnoses (N = 56)

Clinical misdiagnoses	Number	%
**Infections**		
Onychomycosis	13	23.2
Verrruca	11	19.6
Mycosis	3	5.4
Bacterial nail infection	1	1.8
Genital mycosis	1	1.8
**Traumas and lesions**		
Subungual hematoma	2	2.2
Nail bed injury with scar	1	1.8
Nail injury with nail bed inflammation	1	1.8
Skin maceration	1	1.8
Pressure point	1	1.8
Malum perforans	1	1.8
Ulcer	2	3.6
Ulcus cruris	1	1.8
Insect bite	1	1.8
Clavus	1	1.8
**Nevi and tumors**		
Nevus	2	3.6
Riehl's melanosis	1	1.8
Seborrheic keratosis	3	3.3
Solar keratosis	1	1.8
Benign tumor	2	3.6
Inflamed skin tumor	1	1.8
Foreign body granuloma	1	1.8
Basal cell carcinoma	1	1.8
**Other**		
Recurrent inflammation	1	1.8
Eczema	1	1.8
Diabetic foot	1	1.8
**Total**	**56**	**100**

Table S2: Localization, type and frequency of clinically false-negative diagnoses in acral melanomas (n = 38)

Localization	Number	%	False-negative diagnosis	Number
**subungual (hand)**	9	23.7	Onychomycosis	5
			Bacterial nail infection	1
			Subungual hematoma	1
			Nail bed injury with scar	1
			Recurrent inflammation	1
**subungual (foot)**	11	28.9	Onychomycosis	8
			Subungual hematoma	1
			Nail injury with nail bed inflammation	1
			Skin maceration	1
**plantar**	8	21.1	Verruca plantaris	5
			Mycosis	1
			Malum perforans	1
			Plantar ulcer	1
**all others**	10	26.3	Verruca	3
			Mycosis	2
			Verruca vulgaris	1
			Pressure point	1
			Clavus	1
			Diabetic foot	1
			Benign tumor	1

Table S3: Type and frequency of histopathological false-negative diagnoses (n = 73)

**Histopathological false-negative diagnoses**	**Number**	**%**
Nevus cell nevus	41	56.2
Atypical nevus cell nevus	1	1.4
Dysplastic nevus cell nevus	6	8.2
Blue nevus	2	2.7
Spitz nevus	4	5.5
Spindle cell nevus	2	2.7
Basal cell carcinoma	2	2.7
Granuloma pyogenicum	2	2.7
Fibrosarcoma	1	1.4
Malignant fibrous histiocytoma	1	1.4
Histiocytoma	1	1.4
Melanoma in situ	1	1.4
Squamous cell carcinoma	1	1.4
Benign squamous keratosis	1	1.4
Seborrheic keratosis	1	1.4
Verruca vulgaris	1	1.4
Irritant acanthosis	1	1.4
Giant cell tumor	1	1.4
Nonspecific chronic granulating inflammation	1	1.4
Polypous granulation tissue	1	1.4
Traumatic hemorrhage	1	1.4
**Total**	**73**	**100**

Table S4: Clinical and histopathological characteristics of falsely negative diagnosed (FND) melanoma patients, stratified by acral (n=79) and non-acral melanomas (n=127).

	False-negative diagnosis acral (n=79)	False-negative diagnosis non-acral (n=127)	p-value
**Age**			
Median [yrs] (IQR) at FND	58.6 [47.7; 70.4]	51.5 [37.1; 63.4]	<0.001
Median [yrs] (IQR) at correct diag.	60.6 [51.6; 72.0]	55.8 [41.3; 64.7]	0.003
Median [yrs] (IQR) at death of MM	62.8 [54.8; 72.5]	56.5 [42.4; 67.1]	0.037
**Gender**			
Male	28 (35.4%)	59 (46.5%)	
Female	51 (64.6%)	68 (53.6%)	0.120
**Stage at diagnosis (n=185)**			
I	13 (18.6%)	47 (40.9%)	
II	31 (44.3%)	23 (20.0%)	
III	24 (34.3%)	37 (32.2%)	<0.001
IV	2 (2.9%)	8 (7.0%)	
**Tumor thickness (n=150)**			
Median [mm] (IQR)	3.0 [2.0, 5.0]	1.4 [0.95, 2.9]	
<1.00mm	5 (8.3%)	29 (32.2%)	
1.01- 2.00mm	13 (21.7%)	25 (27.8%)	<0.001
2.01- 4.00mm	18 (30.0%)	23 (25.6%)	
>4.00mm	24 (40.0%)	13 (14.4%)	
**Ulceration**			
No	51 (64.6%)	109 (85.8%)	<0.001
Yes	28 (35.4%)	18 (14.2%)	
**Follow-up time***			
Median [months] (IQR)	57.0 [29.0,137.0]	104.0 [37.0,155.0]	0.119
**Death from melanoma**	41 (51.9%)	41 (32.3%)	0.005
**Recurrences during Follow-up**			
Total	53 (67.1%)	89 (70.1%)	0.652
Satellite/in-transit metastases	30 (38.0%)	34 (26.8%)	0.091
Regional lymph node metastases	31 (39.2%)	40 (31.5%)	0.255
Distant metastases	40 (50.6%)	49 (38.6%)	0.090
**Type of FND**			
Clinical FND	60 (75.9%)	46 (36.2%)	<0.001
Histopathological FND	19 (24.1%)	81 (63.8%)	
**Time delay (months)**			
Median [IQR]			
Total	21.0 [10.0; 31.0]	24.0 [10.0; 51.0]	0.054
Clinical FND	18.0 [8.0; 24.0]	21.5 [7.0; 38.3]	0.663
Histopathological FND	25.0 [13.0; 53.0]	29.0 [13.0; 57.0]	0.676

Abbreviation: IQR Inter-quartile Range, * calculated from date of FND until death or censoring
P-values were calculated using Pearson's chi-square test for categorical variables and Mann-Whitney U test for continuous variables, all statistical tests were performed two-sided.

Table S5: Survival probabilities for recurrence-free and melanoma-specific survival in false-negative diagnoses of melanoma, stratified by acral (n=79) and non-acral melanoma (n=127).

	False-negative diagnosis acral (n=79)	False-negative diagnosis non-acral (n=127)	p-value
**Recurrence-free Survival**			
5-yr. rate (95% CI)	47.3% (36.1; 58.5)	47.5% (37.7; 56.3)	0.901
10-yr. rate (95% CI)	32.3% (21.3; 43.3)	33.4% (25.0; 41.8)	
Median [IQR]	50.0 months [20.0; -]	56.0 months [18.0; -]	
**Melanoma-specific Survival**			
5-yr. rate (95% CI)	66.2% (55.6; 76.8)	85.6% (79.5; 91.7)	0.001
10-yr. rate (95% CI)	46.3% (34.5; 58.1)	71.6% (63.6; 79.6)	
Median [IQR]	107.0 months [49.0; -]	not reached	

Abbreviation: CI Confidence Interval, IQR Inter-quartile Range. Calculated from date of FND until death or censoring
Recurrence-free Survival, χ^2 (1) = 0.015, p=0.901, log-rank test, Melanoma-specific Survival, χ^2 (1) = 10.23, p=0.001, log-rank test. All tests were performed two-sided.

Figure S1: Calculation of years of life lost in patients with false-negative diagnosis of melanoma according to method 1 and method 2

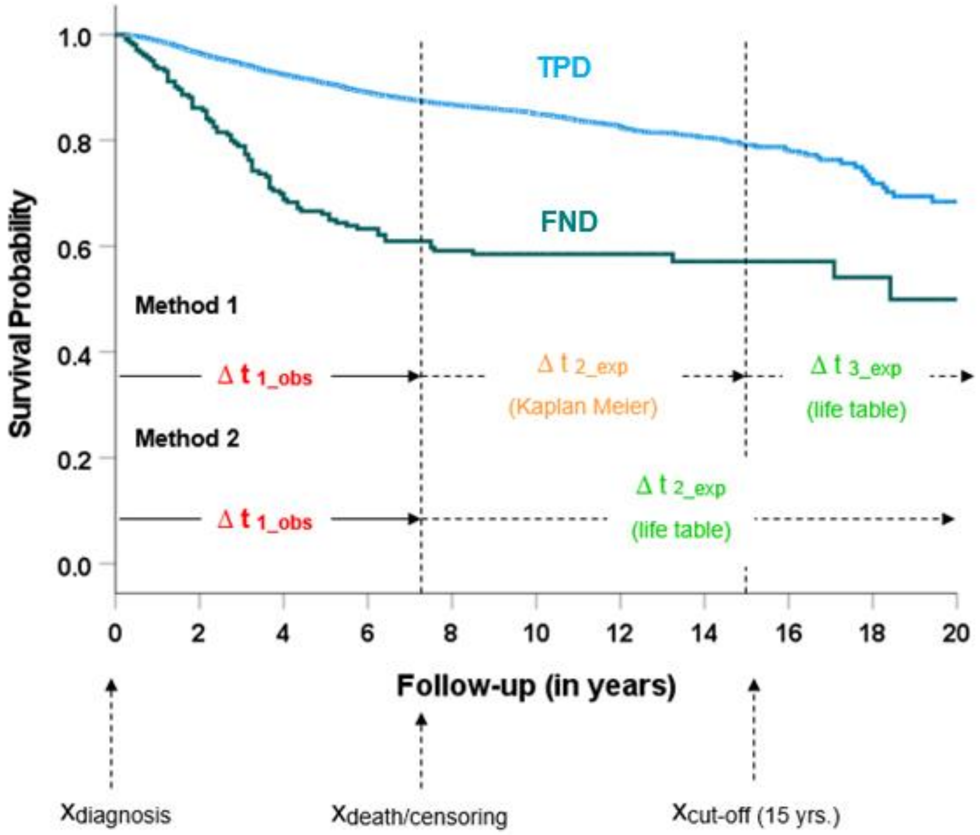

TPD = true-positive diagnosis
FND = false-negative diagnosis

Method 1:

$\triangle t_{1\_obs}$ observed survival time at time point of first diagnosis/FND until death or censoring

$\triangle t_{2\_exp}$ expected survival time (according to Kaplan Meier curve) at time point of censoring until end of observation (cut-off), set to zero for deceased cases

$\triangle t_{3\_exp}$ expected survival time (according to life table) at the end of observation, set to zero for deceased cases

Method 2:

$\triangle t_{1\_obs}$ observed survival time at time point of first diagnosis/FND until death or censoring

$\triangle t_{2\_exp}$ expected survival time (according to life table) at time point of censoring, set to zero for deceased cases
