# Supplementary material for: Years of life lost in patients with a false-negative diagnosis of primary melanoma. A prospective study of the German Central Malignant Melanoma Registry involving 9063 patients over 28 years
Source: Nat Commun. 2026 Jul 2;17:5790. doi: 10.1038/s41467-026-74443-9 (PMC13328647; doi:10.1038/s41467-026-74443-9)
Supplement: Supplementary file 3 — Transparent Peer Review file [file 41467_2026_74443_MOESM3_ESM.pdf]

nature portfolio

Peer Review File

Years of life lost in patients with a false-negative diagnosis of primary melanoma. A prospective study of the German Central Malignant Melanoma Registry involving 9063 patients over 28 years.

Corresponding Author: Professor Claus Garbe

This file contains all reviewer reports in order by version, followed by all author rebuttals in order by version.

Version 0:

Reviewer comments:

Reviewer #1

(Remarks to the Author)
This work attempts to quantify years of life lost following a false negative (clinical or path) diagnosis of melanoma. The authors rightly acknowledge that this information and related data may be used in malpractice suits in future, and so due caution should be taken when interpreting results. A few comments that I believe are critical to consider in this respect:

1. Path FND is difficult / not possible to accurately quantify in this cohort.
If the correct biopsy technique (complete excisional Bx) is taken, then even if the lesion is incorrectly reported benign by the pathologist, the lesion has essentially been removed and is low risk for recurrence (95%+ of primary melanomas are cured with surgery alone)
Therefore, we only know about FND cases that do recur - therefore the true effect of histopath FND is likely overstated.
This is only very briefly acknowledged in the discussion - the impact of this on results / YLL estimations should be discussed.

Of the histopath FNDs – how many were completely excision vs partial sampled with the initial biopsy (partial punch / shave Bx)? If the majority were completely excisised and recurred distantly rather than locally within the scar, it could be argued that the histopath FND did not have a major impact on the course of the disease (ie. metastasis would have occurred regardless of whether a wide local excision was performed if the correct Dx had been made).

2. It is unclear how an assessment of clinical FND was made.
Although registry data was collected prospectively collected, it seems that data relating to the type of FND (clinical vs path), doctor involved and perhaps other details was collected retrospectively via case review. How was clinical FND actually determined? What questions were asked / evidence gathered?
Unless specific questions had been asked of the patient – eg. how many times did you attend for review of the lesion before it was removed it is difficult to extract this type of data from medical records alone.
It is not an uncommon scenario in practice that a patient will report that they sought advice about a lesion from a few different doctors prior to definitive biopsy - this is not necessarily documented in medical records. Work relating to this has been published and may be useful to reference - DOI: 10.1111/ajd.12416 - in this study only 43% of NM were biopsied at a first encounter compared to 70% of SSM

'Clinical FND were mostly revised due to clinical changes of the lesion, to further growth or due to the development of loco-regional or distant metastasis'

It can be difficult to pinpoint the exact point of malignant transformation in melanomas that arise in pre-existing benign naevi. Again, more detail required on how clinical FND was determined.

3. Subtype representation.

Clinical FND were strongly associated with anatomic location of the melanoma; 60 of 106 occurred on acral sites (56.6%) compared to 539 in 8,857 TPD patients (6.1%).
It would be of interest to know what proportion of FND tumors were amelanotic / hypomelanotic. Although it is well recognised that acral lesions can be challenging to diagnose clinically and mimc other benign entities, they are relatively less common than NM (a subtype which is also challenging to diagnose due to a high proportion being hypomelanotic).

Naevoid and desmoplastics subtypes are also known to be challenging to diganose both clinically and on pathology but data relating to these subtypes are not reported in this cohort.

Acral tumors are also less likely to respond to systemic therapies compared with other subtypes, which may also contribute to poorer survival in the FND group.

4. Growth rate estimation.
The delay in diagnosis by 19 months is associated with an increase in tumor thickness of 1.2 mm.
How was this calculated? There are likely to be differences in growth rate between subtypes and also depending on whether the lesion was in situ or invasive on the initial path FND (see doi.org/10.1016/j.jaad.2014.04.068)

5. The role of patient vs clinician delay has not been discussed.
DOI: 10.1002/1097-0215(20000520)89:3<280::aid-ijc11>3.0.co;2-2, DOI: 10.1097/00008390-200208000-00012

Reviewer #2

(Remarks to the Author)
This is a very interesting study that determines the impact of a false negative diagnosis on the years of life lost by patients with cutaneous melanoma. This analysis is particularly relevant, because the median age at diagnosis for patients with melanoma is much lower than that of most solid tumours, therefore the impact of the number of years of life lost due to a missed diagnosis could be much larger.

Patients and Methods

Lines 119-121: It would be interesting to have some additional information about the Central Malignant Melanoma Registry. Is it an hospital-based or population-based registry?

Line 129-130: the authors excluded patients with a follow up time shorter than 3 months. What is the reason for this? Is excluding patients with a short follow-up time not going to bias the estimates, i.e. overestimate survival because of the exclusion of patients with more aggressive lesions and worst prognosis? I would appreciate to touch upon this point in the discussion, i.e. why that was done and what would be the impact of this.

Line 163: please note that the term “survival rate” is incorrect. Survival is a probability, not a rate, taking value between 0 and 1 (or 0 and 100 if expressed as %). Please replace “survival rate” with “survival probability” or simply with “survival” throughout the text, tables and graphics.

Line 162-165: the authors estimated melanoma-specific survival. Cause-specific survival is routinely estimated in clinical trials, because reliable information on the cause of death is available. However, it is not commonly used in population-based study, because the estimate heavily relies on the accuracy of the cause of death, which may vary over time and between geographical areas (see references 1-3 below).
Misclassification of the cause of death can lead to bias estimates.
Therefore, analysis of cancer registry data usually relies on relative survival methods that do not require cause of death information. Net survival is calculated using life tables of background mortality of the general population, to adjust for the risk of dying from causes other than that disease.
I would suggest to report the limitations of using melanoma-specific survival, if estimates of relative survival could not be produced.

Line 165: the Cox model was used to assess the impact of a FND on the hazard of death due to melanoma, after controlling for confounders. Was the assumption of proportional hazard checked? If so, how? Please report it in the methods.

Line 233: I believe that for “normal life expectancy” the authors refer to the life expectancy of the general population. If so, the authors should clarify, otherwise it would lead the reader to imply that resembles a normal distribution.

Line 265: age at diagnosis was categorised into ≤45 years, 46-65 years, >65 years. Can the authors explain why this cut-off were used. Also, what is the lower bound for age, i.e. are only adults included? If so, is the standard cut-off of 15+ years being applied, or 18+?

Results

Line 270-279: the authors should report on the proportion of missing information as well, particularly for variables such as stage, thickness, level of invasion and histological subtype, for which the proportions are over 10% among FND.

Lines 297-300: the Kaplan-Meyer method is a non-parametric method for survival analysis. As such, it is not possible to build up a model in which to specify the outcome and explanatory variables, or confounders. It is possible to stratify survival estimates by categorical variables using Kaplan-Meyer methods, and produce a log-rank test to test the null hypothesis of no differences between the survival curves.
The authors should clarify if this is what has been done or instead a Cox regression model was run, and the p-values are those related to the Wald test.

Lines 304-305: why did the authors decide to include age as a continuous variable in the Cox model? In line 265, age was categorised in 3 groups, ≤45 years, 46-65 years, >65 years.

Discussion

I believe a limitation of the study is not having information on race/ethnicity. It would have been interesting to look at the racial disparity between FND and TPD, particularly because acral melanomas are more frequently diagnosed among darker skinned populations than fairer skinned population.
Similarly, it would have been interesting to look at the impact of socio-economic status. The lack of such variables could be included in the limitations section.

I would appreciate including a paragraph related to the potential bias of using cause-specific survival in the limitations – see comments to lines 162-165.

References

1. C Percy, E Stanek, 3rd, and L Gloeckler "Accuracy of cancer death certificates and its effect on cancer mortality statistics.", American Journal of Public Health 71, no. 3 (March 1, 1981): pp. 242-250.
2. Colin B. Begg, Deborah Schrag, Attribution of Deaths Following Cancer Treatment, JNCI: Journal of the National Cancer Institute, Volume 94, Issue 14, 17 July 2002, Pages 1044–1045, https://doi.org/10.1093/jnci/94.14.1044
3. Diana Sarfati, Tony Blakely, Neil Pearce, Measuring cancer survival in populations: relative survival vs cancer-specific survival, International Journal of Epidemiology, Volume 39, Issue 2, April 2010, Pages 598–610, https://doi.org/10.1093/ije/dyp392

Version 1:

Reviewer comments:

Reviewer #1

(Remarks to the Author)
The reviewers have addressed the majority of concerns. However, there is one critical point that is inaccurate / misleading. This relates to the assertion that:

'Histopathologic misdiagnosis always leads to a higher frequency of recurrence due to the omission of a safety margin excision and the lack of indication for a sentinel lymph node biopsy, which is only performed in cases of melanoma diagnosis.'

Only a small proportion of invasive melanomas will recur / metastasize, with the risk increasing with thickness. As previously mentioned, we will only ever know about the histopath FND cases that do recur/metastasize. A wide local excision is performed to reduce risk of local recurrence. It does not reduce risk of metastasis.

The site of first metastases for melanoma is to regional lymph nodes (~50%-63), locoregional skin / in transit (~13-22%), distant (~24-28%) (Garbe et al. 2002, Adler et al. 2017).

Sentinel node biopsy is a staging procedure considered for melanomas >1mm thickness or 0.8-1mm with other poor prognosis features to detect early LN metastasis. Patient factors such as age and comorbidities factor into a decision about whether SLNB is performed. Therefore, not all people with an invasive melanoma will have SLNB.

Some thin melanomas that do not reach the threshold for SLNB will metastasize.

Furthermore, uptake of SLNB was variable in the period of this review (1996-2015), with MSLT-I (NEJM 2014) showing no survival benefit of the intervention and the post hoc analysis showing potential survival benefit in SLN+ group. Authors will also be aware of the false negative rate for SLNB.

Therefore, it cannot be assumed that ALL people who eventually developed metastases following a false negative histopath diagnosis a) would have had SLNB if a correct diagnosis had been made AND b) would have had a lower frequency of metastasis if WLE+SLNB procedures been undertaken. It is true however, that for some, lymph node metastasis would have been diagnosed sooner (and therefore considered metastatic at diagnosis rather than 'recurrent') had staging (SLNB) been performed.

Reviewer #2

(Remarks to the Author)
I thank the authors for addressing all comments raised in the previous round. The revisions have improved the clarity and quality of the manuscript. I have no further suggestions.

Open Access This Peer Review File is licensed under a Creative Commons Attribution 4.0 International License, which permits use, sharing, adaptation, distribution and reproduction in any medium or format, as long as you give appropriate credit to the original author(s) and the source, provide a link to the Creative Commons license, and indicate if changes were made.
In cases where reviewers are anonymous, credit should be given to 'Anonymous Referee' and the source.
The images or other third party material in this Peer Review File are included in the article's Creative Commons license, unless indicated otherwise in a credit line to the material. If material is not included in the article's Creative Commons license and your intended use is not permitted by statutory regulation or exceeds the permitted use, you will need to obtain permission directly from the copyright holder.
To view a copy of this license, visit https://creativecommons.org/licenses/by/4.0/

Years of life lost in patients with a false-negative diagnosis of primary melanoma.
A prospective study of the German Central Malignant Melanoma Registry involving 9063 patients over 28 years.

Point-by-point response to the reviewers' comments, reproduced verbatim

We would like to thank the reviewers for their critical comments, which we consider helpful and fair. We would like to respond to all critical remarks and comments point by point below.

Reviewer #1 (Remarks to the Author):

This work attempts to quantify years of life lost following a false negative (clinical or path) diagnosis of melanoma. The authors rightly acknowledge that this information and related data may be used in malpractice suits in future, and so due caution should be taken when interpreting results. A few comments that I believe are critical to consider in this respect:

1A. Path FND is difficult / not possible to accurately quantify in this cohort.

If the correct biopsy technique (complete excisional Bx) is taken, then even if the lesion is incorrectly reported benign by the pathologist, the lesion has essentially been removed and is low risk for recurrence (95%+ of primary melanomas are cured with surgery alone). Therefore, we only know about FND cases that do recur - therefore the true effect of histopath FND is likely overstated. This is only very briefly acknowledged in the discussion - the impact of this on results / YLL estimations should be discussed.

This description is entirely accurate. In our data set, approximately 85% of all patients were cured by primary excision alone. This group naturally has an excellent prognosis. However, this group is irrelevant to our analysis because we cannot identify these patients. We can only detect histopathologic misdiagnoses on the basis of tumor recurrence, which is why only patients with tumor recurrence are included in our study. We have now added the following sentences to the discussion:

Histopathologic FNDs could only be detected if tumor recurrence subsequently occurred. All histopathologic FNDs without subsequent tumor recurrence are not included in the analysis. The YLL expectancy would possibly be less if these cases were detectable.

1B. Of the histopath FNDs – how many were completely excision vs partial sampled with the initial biopsy (partial punch / shave Bx)? If the majority were completely excisised and recurred distantly rather than locally within the scar, it could be argued that the histopath FND did not have a major impact on the course of the disease (ie. metastasis would have occurred regardless of whether a wide local excision was performed if the correct Dx had been made).

In fact, complete biopsies of pigmented lesions were performed in more than 95% of patients, as required by German guidelines for decades. However, this does not comply with the guidelines' recommendations for the surgical treatment of melanoma. When melanoma is diagnosed, a re-excision with a safety margin is performed to prevent local recurrence. In patients with a tumor thickness of more than 1 mm, a sentinel lymph node biopsy is also performed, which significantly reduces the risk of recurrence. In this respect, histopathologic FND has an influence on the prognosis. The following statement was included in the discussion:

Histopathologic misdiagnosis always leads to a higher frequency of recurrence due to the omission of a safety margin excision and the lack of indication for a sentinel lymph node biopsy, which is only performed in cases of melanoma diagnosis.

2. It is unclear how an assessment of clinical FND was made.

Although registry data was collected prospectively collected, it seems that data relating to the type of FND (clinical vs path), doctor involved and perhaps other details was collected retrospectively via case review. How was clinical FND actually determined? What questions were asked / evidence gathered?

Unless specific questions had been asked of the patient – eg. how many times did you attend for review of the lesion before it was removed it is difficult to extract this type of data from medical records alone.

It is not an uncommon scenario in practice that a patient will report that they sought advice about a lesion from a few different doctors prior to definitive biopsy - this is not necessarily documented in medical records. Work relating to this has been published and may be useful to reference - DOI: 10.1111/ajd.12416 - in this study only 43% of NM were biopsied at a first encounter compared to 70% of SSM

'Clinical FND were mostly revised due to clinical changes of the lesion, to further growth or due to the development of loco-regional or distant metastasis'

It can be difficult to pinpoint the exact point of malignant transformation in melanomas that arise in pre-existing benign naevi. Again, more detail required on how clinical FND was determined.

We have included a more detailed description of the documentation of FND in the methods section:

When documenting cases from the patient file in the CMMR data set, the medical documentation assistant decided whether a primary FND was present based on the anamnestic information and documented it prospectively. This was only answered with "yes" if the information clearly indicated this. Furthermore, the date of the primary FND was also documented prospectively. Additional data on the FND, such as the exact type of diagnosis and the specialty of the diagnosing physician, were later collected retrospectively from the patient files.

We have also added a statement to the discussion:

In clinical FND, it cannot be completely ruled out that the lesion was still benign at the time of the initial diagnosis and only later became malignant. However, this probability is very low and

cannot be evaluated more precisely. Two-thirds of all primary melanomas arise de novo and are not developed from pre-existing nevi; these are malignant from the very beginning.1-3

1. Duman, N.*, et al.* Nevus-Associated versus de novo Melanoma: Do They Have Different Characteristics and Prognoses? *Dermatopathology (Basel)* **2**, 46-51 (2015).
2. Lai, M., Piana, S., Pellacani, G., Longo, C. & Pampena, R. Prevalence and clinical-pathological features of nevus-associated versus de novo melanoma: a retrospective cross-sectional study of 2806 cases. *Dermatol Pract Concept* **12**, e2022094 (2022).
3. Lin, W.M.*, et al.* Outcome of patients with de novo versus nevus-associated melanoma. *J Am Acad Dermatol* **72**, 54-58 (2015).

3. Subtype representation.

Clinical FND were strongly associated with anatomic location of the melanoma; 60 of 106 occurred on acral sites (56.6%) compared to 539 in 8,857 TPD patients (6.1%).

It would be of interest to know what proportion of FND tumors were amelanotic / hypo-melanotic. Although it is well recognized that acral lesions can be challenging to diagnose clinically and mimic other benign entities, they are relatively less common than NM (a subtype which is also challenging to diagnose due to a high proportion being hypomelanotic).

Naevoid and desmoplastic subtypes are also known to be challenging to diganose both clinically and on pathology but data relating to these subtypes are not reported in this cohort.

Acral tumors are also less likely to respond to systemic therapies compared with other subtypes, which may also contribute to poorer survival in the FND group.

We have only included histological characteristics that were standard in the 1990s. This did not include amelanotic/hypomelanotic information or subtypes of nevoid or desmoplastic melanoma. We are therefore unable to provide any information on these characteristics. We have included a comment on the lower response rates for acral melanoma in the discussion:

Another factor contributing to the poorer outcome of acral melanoma is the lower response rates to immunotherapy and targeted therapies in the metastatic stages.

0. Growth rate estimation.

The delay in diagnosis by 19 months is associated with an increase in tumor thickness of 1.2 mm.

How was this calculated? There are likely to be differences in growth rate between subtypes and also depending on whether the lesion was in situ or invasive on the initial path FND (see doi.org/10.1016/j.jaad.2014.04.068)

To describe the method of calculation, we have amended the paragraph in the discussion as follows:

The 19-month delay in diagnosis for clinical FND is associated with an increase in median tumor thickness from 0.89 mm (TPD) to 2.11 mm (FND) (Table 1), i.e., an increase of 1.2 mm. The growth rate of the primary melanoma can therefore be considered slow.

1. The role of patient vs clinician delay has not been discussed.

DOI: 10.1002/1097-0215(20000520)89:3<280::aid-ijc11>3.0.co;2-2, DOI: 10.1097/00008390-200208000-00012

To cover this topic, we have added a paragraph to the discussion and cited the publications suggested by the reviewer:

Medical FND causes a delay in diagnosis in a small percentage of patients. In comparison, the delay in diagnosis by patients themselves is considered to be more significant. Approximately one-third of all patients wait more than a year to see a doctor after observing the first signs of melanoma.

*Schmid-Wendtner, M.H., Baumert, J., Stange, J. & Volkenandt, M. Delay in the diagnosis of cutaneous melanoma: an analysis of 233 patients. Melanoma Res **12**, 389-394 (2002).*

*Richard, M.A., et al. Delays in diagnosis and melanoma prognosis (I): the role of patients. Int J Cancer **89**, 271-279 (2000).*

*Richard, M.A., et al. Delays in diagnosis and melanoma prognosis (II): the role of doctors. Int J Cancer **89**, 280-285 (2000).*

Reviewer #2 (Remarks to the Author):

This is a very interesting study that determines the impact of a false negative diagnosis on the years of life lost by patients with cutaneous melanoma. This analysis is particularly relevant, because the median age at diagnosis for patients with melanoma is much lower than that of most solid tumours, therefore the impact of the number of years of life lost due to a missed diagnosis could be much larger.

Patients and Methods

2. Lines 119-121: It would be interesting to have some additional information about the Central Malignant Melanoma Registry. Is it an hospital-based or population-based registry?

We added to Methods:

The CMMR is a hospital-based registry. Approximately 60 departments in Germany have documented their patients on a voluntary basis without remuneration. Demographic data, tumor data, basic data on treatment, follow-up and the course of the disease were recorded.

3. Line 129-130: the authors excluded patients with a follow up time shorter than 3 months. What is the reason for this? Is excluding patients with a short follow-up time not going to bias the estimates, i.e. overestimate survival because of the exclusion of patients with more aggressive lesions and worst prognosis? I would appreciate to touch upon this point in the discussion, i.e. why that was done and what would be the impact of this.

We added to Discussion:

Patients who had a follow-up time of at least 3 months were included in this study. Our intention was to distinguish between patients with and without follow-up, as the shortest time interval according to German guidelines is three months. The probability of dying from melanoma in the first three months after the initial diagnosis is very low. This approach was also used in other CMMR studies.

Garbe, C., et al. Prognosis of Patients With Primary Melanoma Stage I and II According to American Joint Committee on Cancer Version 8 Validated in Two Independent Cohorts: Implications for Adjuvant Treatment. J Clin Oncol 40, 3741-3749 (2022).
Garbe, C., et al. Prognosis of Patients With Stage III Melanoma According to American Joint Committee on Cancer Version 8: A Reassessment on the Basis of 3 Independent Stage III Melanoma Cohorts. J Clin Oncol 38, 2543-2551 (2020).
Teramoto, Y., et al. Acral lentiginous melanoma: a skin cancer with unfavourable prognostic features. A study of the German central malignant melanoma registry (CMMR) in 2050 patients. Br J Dermatol 178, 443-451 (2018).

4. Line 163: please note that the term "survival rate" is incorrect. Survival is a probability, not a rate, taking value between 0 and 1 (or 0 and 100 if expressed as %). Please replace "survival rate" with "survival probability" or simply with "survival" throughout the text, tables and graphics.

We replaced the term "survival rates" with "survival probabilities" (8 times).

5. Line 162-165: the authors estimated melanoma-specific survival. Cause-specific survival is routinely estimated in clinical trials, because reliable information on the cause of death is available. However, it is not commonly used in population-based study, because the estimate heavily relies on the accuracy of the cause of death, which may vary over time and between geographical areas (see references 1-3 below).

Misclassification of the cause of death can lead to bias estimates.

Therefore, analysis of cancer registry data usually relies on relative survival methods that do not require cause of death information. Net survival is calculated using life tables of background mortality of the general population, to adjust for the risk of dying from causes other than that disease.

I would suggest to report the limitations of using melanoma-specific survival, if estimates of relative survival could not be produced.

We agree with the reviewer that the collection of death data and causes of death is crucial for an analysis of melanoma-specific survival. The CMMR is not a population-based registry, but a hospital-based registry. This study only includes data from the University Hospital of Tübingen. Here, we have established a strategy for recording death data and causes of death, which is described below and included in the methods section:

An active approach to recording death data and causes of death for the CMMR has been established at the University Hospital of Tübingen. If follow-up was discontinued, the family, the treating physicians, or even the residents' registration offices were contacted for information.

0. Line 165: the Cox model was used to assess the impact of a FND on the hazard of death due to melanoma, after controlling for confounders. Was the assumption of proportional hazard checked? If so, how? Please report it in the methods.

Thank you for this important note.

We added a paragraph in the "Methods" section addressing the assessment of the proportional hazard assumption for the Cox regression model:

The assumption of proportional hazards was evaluated by visual inspection and testing of Schoenfeld residuals. Within a period of 120 months, the standard follow-up time for melanoma in Germany, no violation of the proportional hazards' assumption was found (global test: p = 0.85).

1. Line 233: I believe that for "normal life expectancy" the authors refer to the life expectancy of the general population. If so, the authors should clarify, otherwise it would lead the reader to imply that resembles a normal distribution.

The sentence has now been supplemented as follows:

Method 2 is based on the assumption that patients have a normal life expectancy at the time point of censoring based on established Generation Life Tables for Germany.

2. Line 265: age at diagnosis was categorised into ≤45 years, 46-65 years, >65 years. Can the authors explain why this cut-off were used. Also, what is the lower bound for age, i.e. are only adults included? If so, is the standard cut-off of 15+ years being applied, or 18+?

We included patients of all ages, including a few under the age of 18 (0.5%).

3. Line 270-279: the authors should report on the proportion of missing information as well, particularly for variables such as stage, thickness, level of invasion and histological subtype, for which the proportions are over 10% among FND.

We have added the proportion of missing data to Table 1. We have also added a paragraph to the "Results" section describing the proportion of missing data:

Incomplete data, particularly in the group of FND patients, were found for stage at initial diagnosis (10.2%), tumor thickness (27.2%), level of invasion (35.4%), and for histological subtype (15.1%).

4. Lines 297-300: the Kaplan-Meyer method is a non-parametric method for survival analysis. As such, it is not possible to build up a model in which to specify the outcome and explanatory variables, or confounders. It is possible to stratify survival estimates by categorical variables using Kaplan-Meyer methods, and produce a log-rank test to test the null hypothesis of no differences between the survival curves.

The authors should clarify if this is what has been done or instead a Cox regression model was run, and the p-values are those related to the Wald test.

Statistical testing for survival curves according to Kaplan Meier was performed by the log-rank test. We have supplemented this now in the Results section:

Further Kaplan-Meier analyses of MSS in the total sample (FND and TPD) showed that age at first diagnosis, AJCC stage, tumor thickness, Clark level of invasion, histopathological subtype, anatomic location of primary tumor, localization on acral sites (all p<0.001), gender, (p=0.014) and ulceration (p=0.009) were significant prognostic factors in univariate analysis, based on the results of the log-rank test (data not shown).

The reported p-values within the Cox model are from the Wald tests. This has now been clarified in the "Methods" section

The reported p-values are from the Wald test within the Cox model.

as well as in the “Results”:

All factors included in the model were identified as significant prognostic factors for MSS ($p < 0.05$; Wald Test).

6. Lines 304-305: why did the authors decide to include age as a continuous variable in the Cox model? In line 265, age was categorised in 3 groups, ≤45 years, 46-65 years, >65 years.

We have now calculated two different Cox models, one with age as a continuous variable and another with age as a categorical variable. We compared both models using the AIC criterion. Based on the results of the AIC values (age continuous: AIC=20085,5 vs. age categorical: AIC=20128,4), we decided to present the Cox model with age as a continuous covariate.

Discussion

7. I believe a limitation of the study is not having information on race/ethnicity. It would have been interesting to look at the racial disparity between FND and TPD, particularly because acral melanomas are more frequently diagnosed among darker skinned populations than fairer skinned population.

The study population was exclusively included in Germany, and people of color are rare in Germany. The black patients I have seen in my professional career (CG) were usually American soldiers stationed in Germany who have their own medical care. We have guest workers, usually from Mediterranean countries, who are ethnically Caucasian. Therefore, subgroup analyses for race/ethnicity cannot be performed.

8. Similarly, it would have been interesting to look at the impact of socio-economic status. The lack of such variables could be included in the limitations section.

We have added the following sentence under Limitations:

Unfortunately, we did not have data on socioeconomic status, so we were unable to analyze its impact on FND.

9. I would appreciate including a paragraph related to the potential bias of using cause-specific survival in the limitations – see comments to lines 162-165.

We have added the following sentence under Limitations:

The endpoint of melanoma-specific survival depends on the quality of the data on deaths and causes of death. Despite active strategies to collect this data, we are unlikely to achieve the same quality for this endpoint as in prospective randomized studies.
